# Supplementary material for: Proteome-scale tissue mapping using mass spectrometry based on label-free and multiplexed workflows
Source: bioRxiv. 2024 Mar 4:2024.03.04.583367. Preprint. [Version 1] doi: 10.1101/2024.03.04.583367 (PMC10942300; doi:10.1101/2024.03.04.583367)
Supplement: Supplementary file 1 [file media-1.pdf]

Supplementary information for:

Proteome-scale tissue mapping using mass spectrometry based on label-free and multiplexed workflows

Yumi Kwon,[1] Jongmin Woo,[1,§] Fengchao Yu,[2] Sarah M. Williams,[1] Lye Meng Markillie,[1] Ronald J. Moore,[3] Ernesto S. Nakayasu,[3] Jing Chen,[4] Martha Campbell-Thompson,[4] Clayton E. Mathews,[4] Alexey I. Nesvizhskii,[2,5] Wei-Jun Qian,[3,*] and Ying Zhu [6,*]

[1]Environmental Molecular Sciences Laboratory, Pacific Northwest National Laboratory, Richland, WA 99354, United States

[2]Department of Pathology, University of Michigan, Ann Arbor, MI 48109, United States

[3]Biological Sciences Division, Pacific Northwest National Laboratory, Richland, WA 99354, United States

[4]Department of Pathology, Immunology and Laboratory Medicine, University of Florida, Gainesville, FL 32610, United States

[5]Department of Computational Medicine and Bioinformatics, University of Michigan, Ann Arbor, MI 48109, United States

[6]Department of Microchemistry, Proteomics, Lipidomics and Next Generation Sequencing, Genentech, 1 DNA Way, South San Francisco, CA 94080, United States

[§]Current address: Department of Pathology, Johns Hopkins University School of Medicine, Baltimore, MD 21287, United States

*Corresponding authors:

Dr. Ying Zhu, Email: zhu.ying@gene.com

Dr. Wei-Jun Qian, Email: Weijun.Qian@pnnl.gov

Table of Contents:

Title		Page
**Supplementary Figure 1**	Optimization of gradient time for TIFF method.	3
**Supplementary Figure 2**	Evaluation of quantification performance of TIFF method using two-species proteome.	4
**Supplementary Figure 3**	Proteome identification number by the TIFF method in label free approach.	5
**Supplementary Figure 4**	Proteome identification number in TMT method	6
**Supplementary Figure 5**	Pearson correlation matrix for the pure islet and acinar pixels.	7
**Supplementary Figure 6**	UMAP plot for each islet pair and quantification method.	8
**Supplementary Figure 7**	Comparison of transcriptomic markers identified in human pancreatic tissue and the proteome markers.	9
**Supplementary Figure 8**	Summary of quantification methods for high-resolution spatial proteomics study.	10

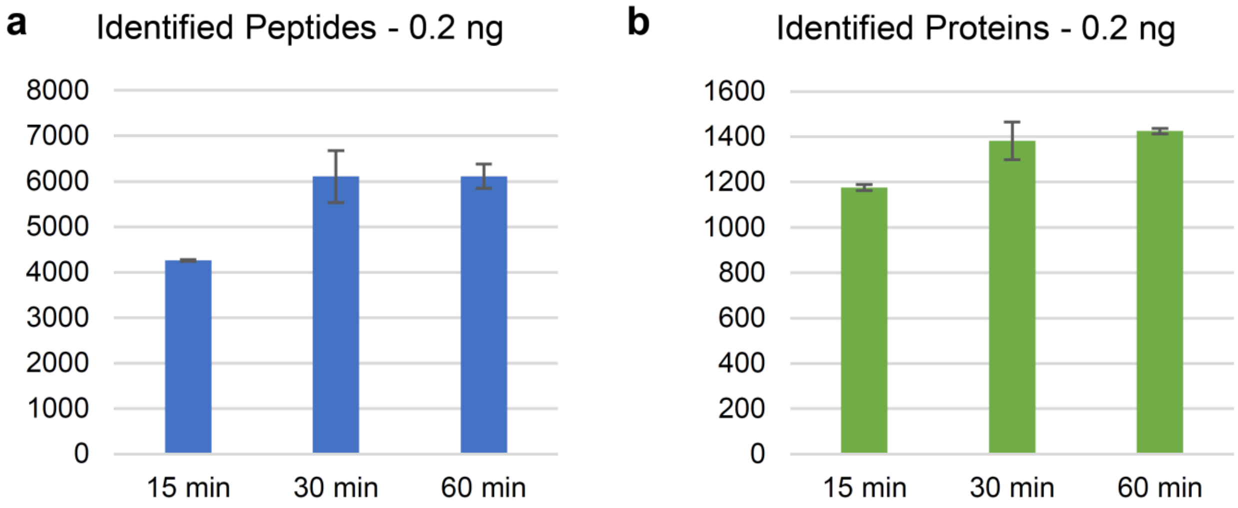

Supplementary Figure 1. Optimization of gradient time for TIFF method. (**a**) Number of identified peptides and (**b**) proteins at three LC gradient times (15, 30 and 60 min). A model sample of 0.2 ng S. Oneidensis peptide was used.

a

	M. musculus	*S. Oneidensis*
Spectral Library	10 ng	3 ng
Run 1	0.1 ng	0.01 ng
Run 2	0.1 ng	0.03 ng

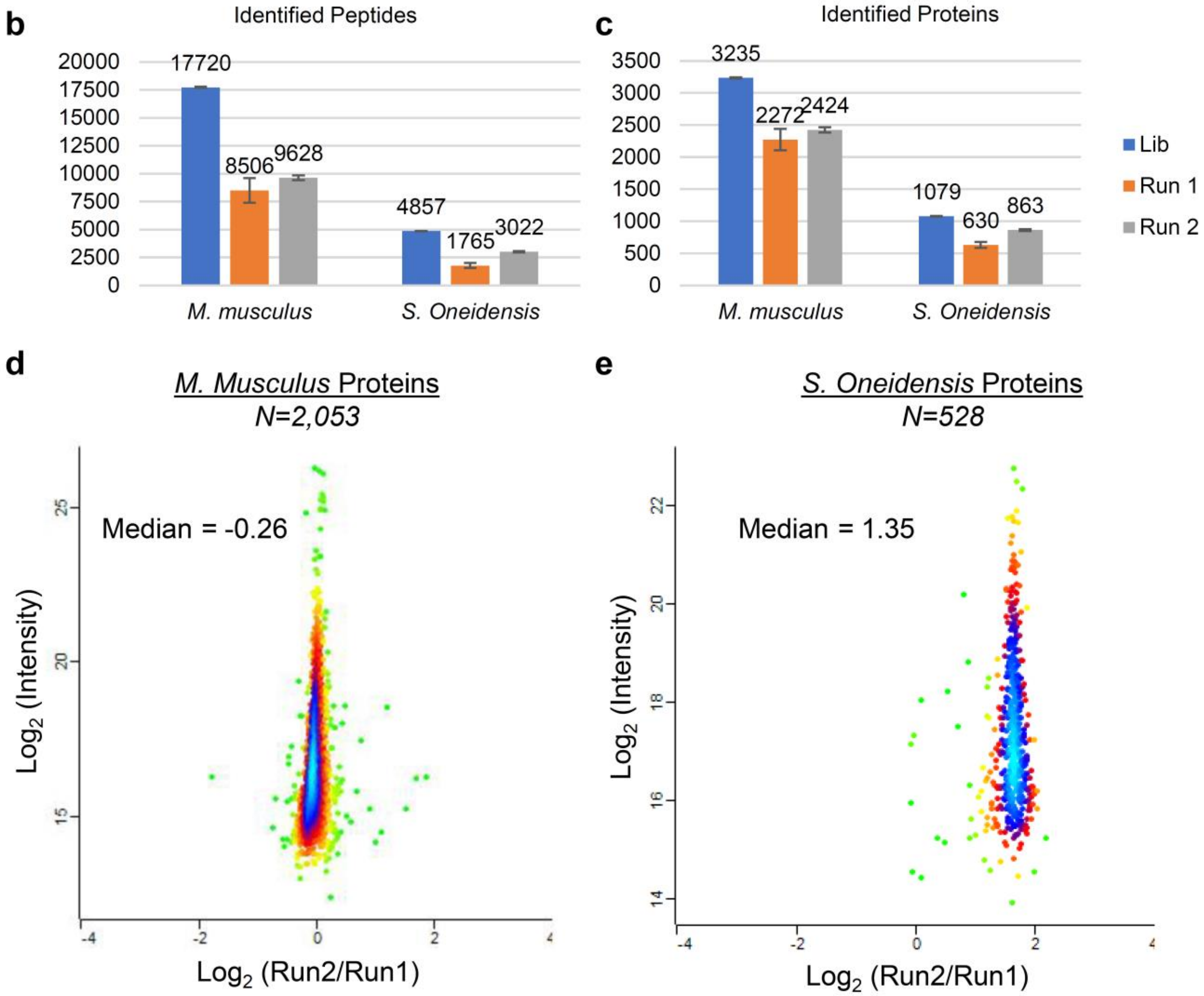

Supplementary Figure 2. Evaluation of quantification performance of TIFF method using two-species proteome. (**a**) Experimental design of the two-proteome study including the spectral library, Run 1, and Run 2. (**b**) Peptides and (**c**) proteins identified from two proteome mixture in library, run 1 and run 2, respectively. (**d**) Scatter plots showing protein fold changes between Run2 and Run1 for mouse proteome and (**e**) Shewanella proteome.

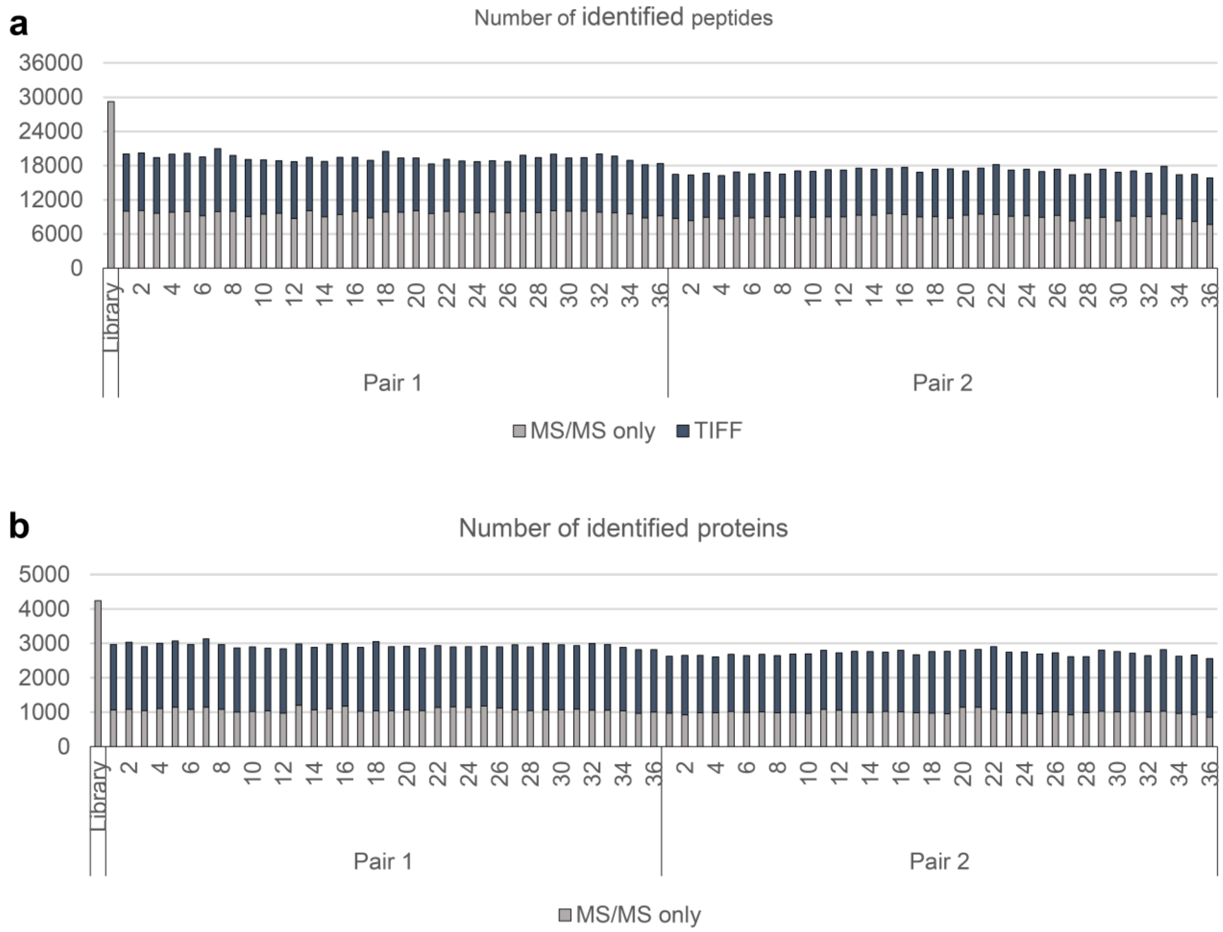

Supplementary Figure 3. Number of peptides (**a**) and protein groups (**b**) in single pixels identified by MS/MS only (gray) and by the TIFF method (blue) in label free approach.

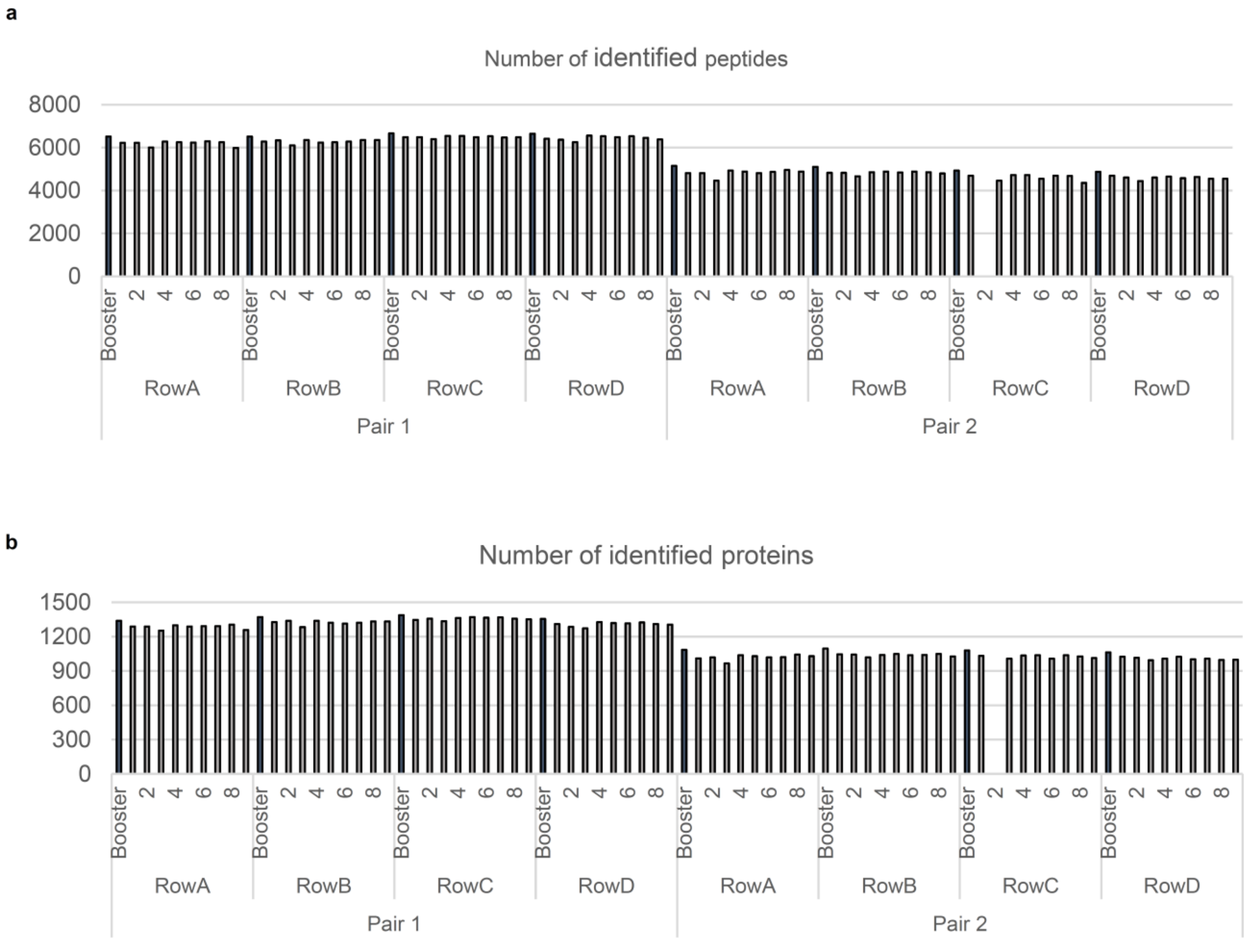

Supplementary Figure 4. Number of peptide (**a**) and protein groups (**b**) in single pixels using multiplexed TMT approaches**.**

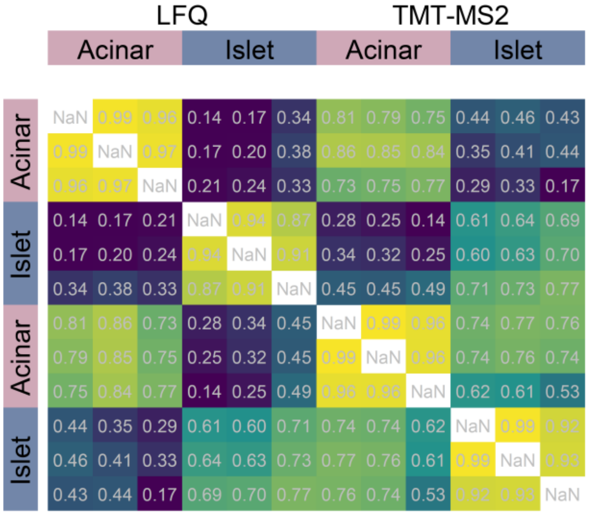

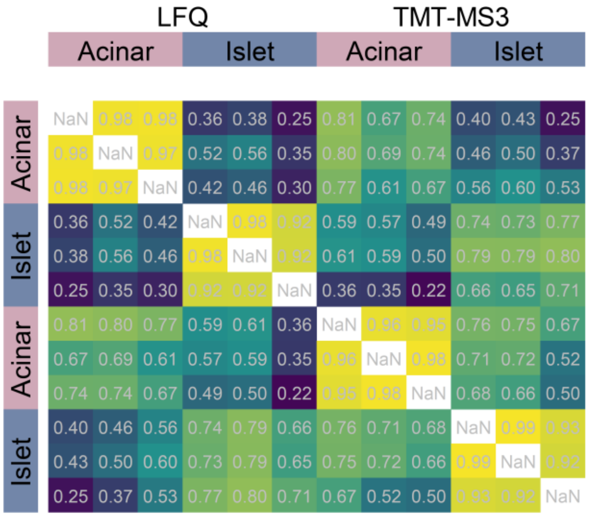

**0.97**	**0.24**	**0.79**	**0.37**
**0.24**	**0.91**	**0.33**	**0.68**
**0.79**	**0.33**	**0.97**	**0.69**
**0.37**	**0.68**	**0.69**	**0.95**

**0.98**	**0.40**	**0.72**	**0.46**
**0.40**	**0.94**	**0.48**	**0.74**
**0.72**	**0.48**	**0.97**	**0.66**
**0.46**	**0.74**	**0.66**	**0.95**

Supplementary Figure 5. Pearson correlation matrix for the pure islet and acinar pixels. Correlation of protein abundances between cell-types and quantification methods are shown in the correlation matrix. Median values of correlation coefficients for each cell type and quantification method were shown in the bottom panel.

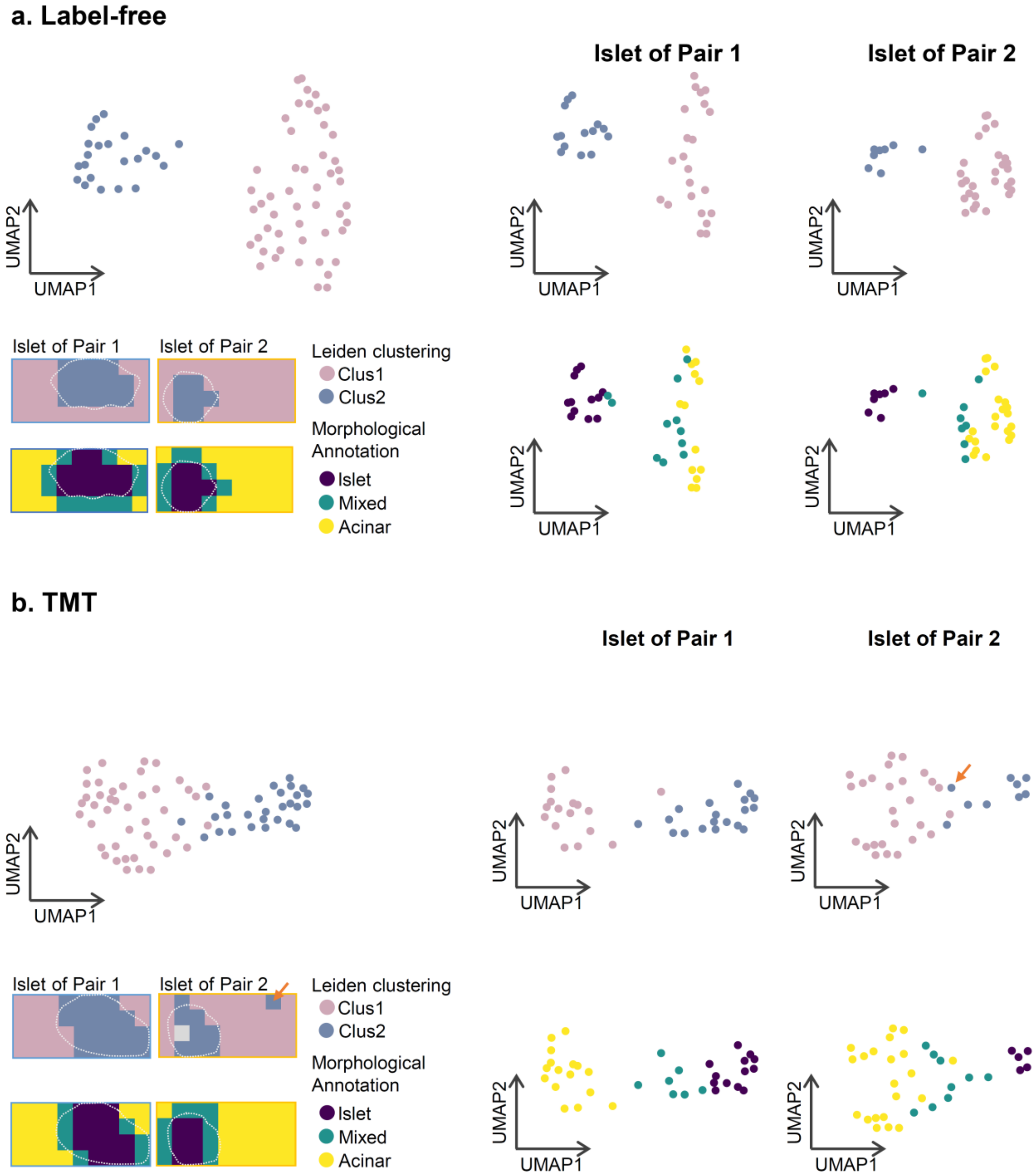

Supplementary Figure 6. UMAP plot for each islet pair and quantification methods for (a) label free and (b) TMT methods.

a

b

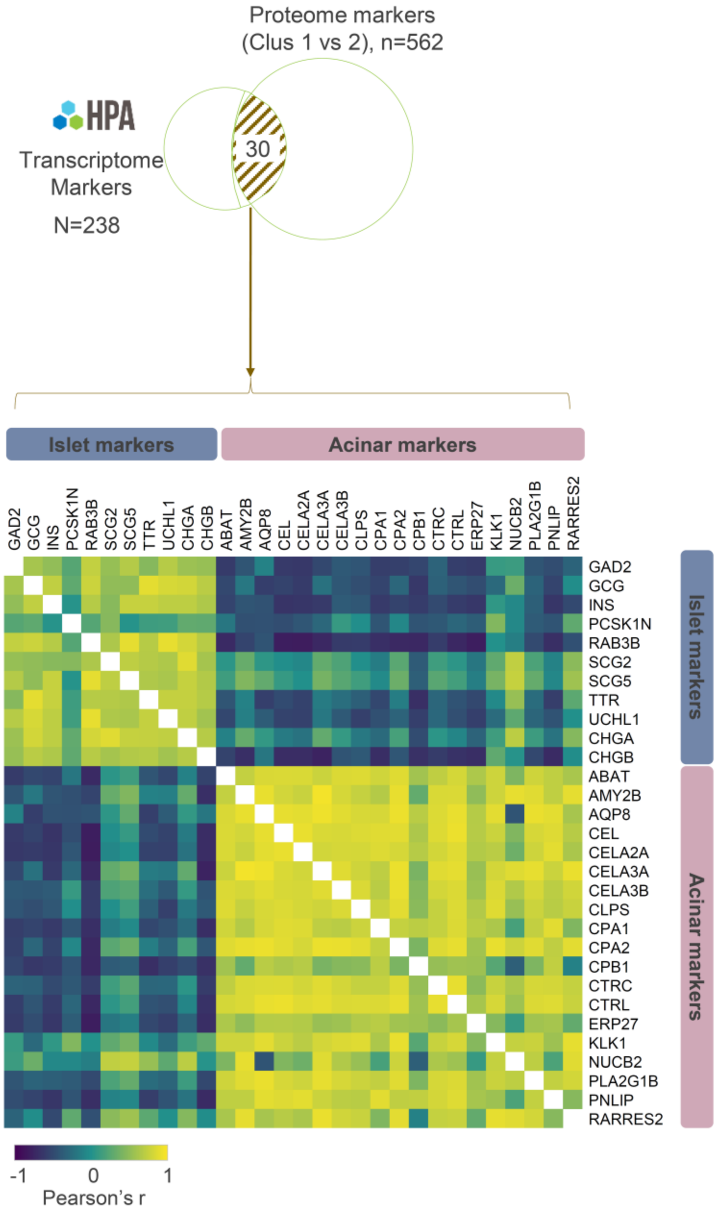

Supplementary Figure 7. (**a**) Venn diagram indicating the overlap of transcript markers and protein markers identified from Human Protein Atlas (HPA) database and this study, respectively. (**b**) Pearson correlation of commonly identified pancreatic markers.

a

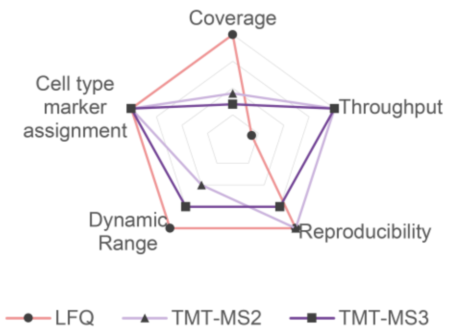

b

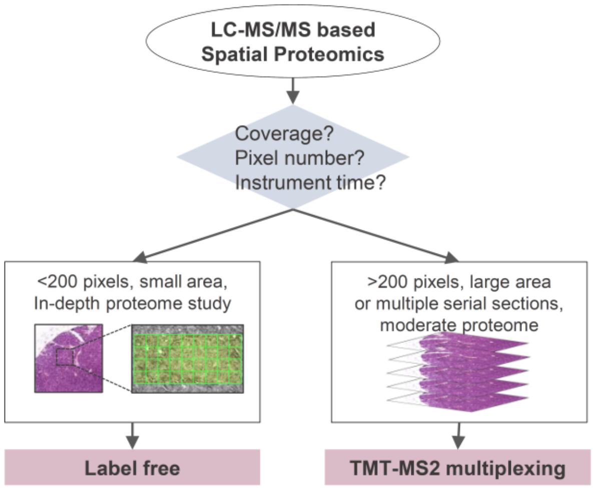

c

Method	Throughput	Number of quantifiable IDs	Dynamic Range	Cell type marker assignment
LFQ	+	+++	+++	+++
TMT-MS2	+++	++	+	++
TMT-MS3	+++	+	+	++

Supplementary Figure 8. Summary of quantification methods for high-resolution spatial proteomics study. (**a**) Radar chart for relative comparison of three quantification approaches, including label-free quantification (LFQ), TMT-MS2, and TMT-MS3 analysis based on coverage, sample throughput, reproducibility, dynamic range, and performance for cell-type marker assignment. From the inside to the outside, the outer point represents better performance for a given trait. (**b**) Flow chart for making a decision of the most suitable quantification method for high-resolution proteome mapping based on experimental design. (**c**) A summary table showing the performance of the three quantification approaches.
